# Public health nurses’ experiences working with children who are next of kin: a qualitative study

**DOI:** 10.1186/s12913-022-08841-2

**Published:** 2022-11-28

**Authors:** Marie Dahlen Granrud, Tuva Sandsdalen, Agneta Anderzén-Carlsson, Anne Kjersti Myhrene Steffenak

**Affiliations:** 1 Faculty of Health and Social Sciences, Inland Norway University of Applied Sciences, PB 400, 2418 Elverum, Norway; 2 University Health Care Research Centre, Faculty of Medicine and Health, Örebro University, Örebro, Sweden

**Keywords:** Child, Child health clinic, Children who are next of kin, Community health, Public health nurse, School health service

## Abstract

**Background:**

There are a substantial number of children who are the next of kin of parents suffering from illness or substance abuse. These children can experience emotional and behavioral problems and may need support from professionals. In Norway, the specialist health service in hospitals is required to have a designated practitioner in each department to ensure support for and follow up of children who are next of kin; however, this is not regulated by law in the health care in the municipalities. The aim of this study was to explore public health nurse’s experiences working with children who are next of kin.

**Methods:**

Qualitative interviews were conducted with 10 public health nurses working in the child health clinic and the school health service in four municipalities. Data were analysed using content analysis. Reporting of this study is conducted in accordance to COREQ’s checklist.

**Results:**

The analysis resulted in one main theme: ‘Lack of guidelines and routines among public health nurses working with children who are next of kin’. The main theme consisted of four categories: (1) identifying children who are next of kin are incidental; (2) public health nurses must be observant and willing to act; (3) communication is an important tool; and (4) follow up over time is not always provided.

**Conclusion:**

The public health nurses experienced uncertainty concerning how to identify and follow up children who are next of kin but were vigilant and willing to act in the children’s best interest. Doing so necessitated collaboration with other professionals. The need for guidelines around the role and responsibilities for the public health nurse were emphasized. The knowledge provided by the current study offers valuable insight into strengths and limitations in the support of children who are next of kin and can inform stakeholders in organizing sustainable support for this group.

## Background

Children who are next of kin include children under 18 years with parents, adoptive parents, stepparents or foster parents suffering from serious somatic illness or injury, mental disease or substance abuse [1]. Worldwide, the prevalence of children who are next of kin is reported at 2–8% [2, 3], though this number differs depending on the definition being used [3]. A multi-centre study in Norway has estimated that, every year, about 350,000 children are next of kin to parents with different diagnosis receiving health care from the specialist health services [4]. As it is assumed that not all children who are next of kin are identified, these numbers are expected to be higher.

Children who are next of kin of parents with mental illness can experience emotional and behavioural problems as well as anxiety and depression [1, 5, 6]. They are at a higher risk of developing mental and social problems than other children and experience conflict-filled family interactions, poorer parental functioning and neglect [7, 8]. Previous research has found that children who are next of kin report unpredictability in their daily life related to worries about the ill parent’s condition. In addition, children whose parents are having problems with substance abuse, report that they are troubled by feelings of guilt [9].

In particular, children who are the next of kin of a parent with mental illness report a lack of close relationships with peers; they report being bullied and laughed at, which leads to feelings of loneliness and sadness [10]. Indeed, their level of worry—which varies according to the parent’s different challenges—may be a product of the stigma and discrimination associated with mental illness [11]. Stigma is reported as one reason families and children are uncomfortable disclosing their situation with mental illness to others [12].

Furthermore, previous research has found that children who have parents with physical illness and disability report higher level of worry about their parents, but less caregiving discomfort than children of parents with mental illness [11]. Golsäter et al. [13] describe that children who are next of kin of parents with cancer need information and knowledge about their parent’s situation. They argue that it is important that the information is based on the ill parent’s specific situation (i.e., their disease, treatment and care) and tailored to the child’s individual needs. When health professionals communicate with children about the parent’s illness, this helps reduce the children’s anxieties about what is happening within their families [14]. Moreover, children who are next of kin might need support from different professionals in the health and social services [15]. Depending on the care burden, their support needs vary; as such, support must be tailored to their individual situation [16].

In Norway, amendments made in 2010 to the Specialist Health Service and Health Personnel Acts stipulate that all hospitals must have a designated practitioner who is responsible for ensuring follow up of children who are next of kin in wards, clinics and institutions [17, 18]. However, this is not required by law in municipal care, within which the child health clinics and the school health service are organized. Nevertheless, as public health nurses (PHNs) work in the municipalities, they can identify children who are next of kin and support them in the context where they live their daily life. PHNs in Norway are registered nurses with at least 1 year of postgraduate education in public health nursing, with expertise in health promotion and disease prevention among children aged 0 to 20 years, and their families. Their task is to monitor, identify, guide, counsel and refer to other professionals when needed [19].

A number of studies exist on children’s perspectives as next of kin, as well as the perspectives of parents [20–22]. Research indicates that children want information and involvement in their parent’s situations [22] and parents want support from health personnel regarding how best to communicate with their children; moreover, parents report a discrepancy between the desired support and what is provided [14]. There are also studies on how nurses and general practitioners [GPs] experience their role regarding identifying, supporting and following up with children who are next of kin in the specialist and primary health services [9, 23–25].

To our knowledge, there is a lack of studies that describe PHNs’ perspective on working with children who are next of kin in the municipal care context were the PHN meet with almost all children. Such knowledge is important as it could offer valuable insight into strengths and limitations in the current support of children who are next of kin and can inform stakeholders in organizing sustainable support for this group. Therefore, the aim of this study was to explore PHN’s experiences working with children who are next of kin.

## Methods

### Design

To gain a deeper understanding of PHNs’ experiences working with children who are next of kin, a qualitative design was used. Data were collected through individual interviews [26] and analysed via qualitative content analysis [27]. The reporting of this study is conducted in accordance to COREQ checklist [28].

### Setting

The setting for this study was the Norwegian child health clinics and school health service, in both rural and urban municipalities. In Norway, PHNs in child health clinics work with children up to five years and their families. There are 14 regular consultations throughout the first 5 years of the child’s life—these occur most frequently during the first 2 years. In the school health service, PHNs work with children aged 5 to 19 years [29]. They have regularly consultations during the 10 first years of school attendance. PHNs who work in secondary schools also strive to have an ‘open door policy’ so that children can visit whenever they want [30].

### Participants

In total, 10 PHNs participated in the study. They worked in child health clinics (*n* = 4) and the school health service (*n* = 6) in four different municipalities in one county. The municipalities varied in size from 3500 to 20 000 inhabitants. Inhabitants in the municipalities have no more than a 30 minutes’ drive to access the nearest hospital. Recruitment was carried out by contacting leaders in different municipalities via mail with information about the study. The leaders were asked to identify PHNs with experiences with children who are next of kin and inform them about the study. The PHNs who wanted to participate contacted the first author and an appointment for the interview was established. Despite repeated requests and a prolonged recruitment period, only 10 PHNs volunteered to participate. They were all women and their age varied from 40 to 56 years (mean 46), with work experience as a PHN ranging from 4 to 20 years. Three of the PHNs had at least one advanced degree. All the PHNs had experience with the topic, having followed up with between 6 and 40 children who are next of kin.

### Data collection

Data were collected between May 2020 and February 2021. A semi-structured interview guide was used to ensure that the aim of the study was covered and that each participant was asked to elaborate on the same topics [26]. All 10 interviews were individual and conducted by the first author. Five interviews were conducted via a digital platform, due to the COVID-19 pandemic. Four took place at the PHNs’ workplace, in accordance with their preferences, and one took place at the first author’s workplace. The interviews lasted from 32 to 52 min, with an average length of 37 min. The interviews were audio recorded and transcribed verbatim. The interview guide included the following main questions: What are your experiences with children who are next of kin? How do you support and follow up children who are next of kin? Follow-up question were asked to obtain more detailed information of the PHNs’ experiences; for example, ‘Can you please tell me more about…?’ and ‘Can you please explain more about that…?’

### Data analysis

The first author carried out the analysis in collaboration with the other authors. The interviews were analysed using qualitative content analysis, as described by Graneheim and Lundman [27]. The interviews were transcribed, and to ensure that they were transcribed correctly and accurately, the recorded interviews were first listened to in their entirety by the first author, while at the same time reading the transcripts. Next, all authors read the transcribed interviews to familiarize themselves with the content and to acquire an overall understanding. Meaning units, which corresponded with the aim of the study, were identified and extracted—these were words or sentences containing aspects that were related to each other through their content or context. The meaning units were then abstracted into codes, which were compared based on similarities and differences; this resulted in categories accurately representing the content of the data [27]. Finally, one theme that unified the content in the categories was formulated. This theme illuminated the underlying meaning from the meaning units, codes and categories at an interpretative level [27].

The first author analysed four interviews and the other three authors were given two interviews each to analyse. Then, these interviews were switched by the authors to ensure agreement in the labelling and content of the categories. All the authors participated in the analysis process and continuously discussed the process. When disagreement arose, the discussion continued until consensus was achieved.

All of the authors are registered nurses, and one has further education in public health nursing. They had experience working with children who are next of kin and conducting research related to this subject. The authors were aware of the possibility that the knowledge gained from their clinical practice, education and previous research on the subject might influence their pre-understandings of the study’s phenomena during the analysis. However, the authors believe that this knowledge contributed to widening and deepening the analysis of the data.

## Results

The data analysis resulted in one main theme: ‘Lack of guidelines and routines among public health nurses working with children who are next of kin’. This theme consisted of four categories (for an overview of the theme and categories, see Table 1). The theme and categories are presented below, and the categories are illustrated by quotations.

**Table 1:** Overview of the theme and categories

Theme	Lack of guidelines and routines among public health nurses working with children who are next of kin
**Categories**	• Identifying children who are next of kin was incidental • Public health nurses must be observant and willing to act • Communication is an important tool • Follow up over time is not always provided

### Lack of guidelines and routines among public health nurses working with children who are next of kin

The analysis pointed to a lack of systematics around working with children who are next of kin. The PHNs in both the child health clinics and the school health service reported that it was purely incidental as to whether they identified a child as being next of kin. This varied, according to the PHNs’ prior experiences with children who are next of kin, the care context and how the PHNs perceived their role. The PHNs in the child health clinics had less experience with children who are next of kin than the PHNs in the school health service. All the PHNs reported being dependent on others to identify children as next of kin. In addition, it differed as to how the PHNs in the child health clinics and the school health service perceived their work related to children who are next of kin. In the child health clinics, the PHNs more often worked indirectly with the children and expressed more uncertainty about their role. This role was a bit clearer in the school health service, where the PHNs worked more directly with the children.

In both settings, the PHNs felt that they needed to be observant to identify children who are next of kin. It was regarded as being up to each PHN as to how to identify the children and what actions to take, and this varied from case to case. Moreover, most of the PHNs regarded communication as an important tool to support children who are next of kin, but there were no clear guidelines. These actions should be performed, which was interpreted as a lack of systematics. Finally, there were no clear guidelines or routines for how children who are next of kin should be followed up over time, or what constitutes their best interest.

#### Identifying children who are next of kin is incidental

As noted above, the PHNs in both the child health clinics and in the school health service described it as incidental as to whether and how children who are next of kin were identified. The PHNs mainly identified these children at regular consultations or through parents and were seldom contacted by the specialist health service. The PHNs expressed certainty that many children who are next of kin are currently unidentified. As one said,

“*To find them, you have to be a detective.*” *(Int. 4).*

The PHNs in the child health clinics and the school health service explained that they typically learned that a child was next of kin through being informed about a parent’s illness. Typically, one of the parents would tell the PHN about the illness—although this was more common when the parent suffered from a physical illness, like cancer, as compared to mental illness. With regards to the latter, the PHNs experienced that the threshold for parental contact was higher. However, one PHN recalled an instance in which a mother contacted her because she needed help—or rather, she believed her child needed support because of the child’s father’s drug addiction:

“*One mother asked for help. She was uncertain what she should do. Otherwise, it’s difficult to detect. You can’t detect this without someone is telling you.” (Int. 9).*

The PHNs in the school health service described how many children visit PHNs for other reasons, and it was during such a visit that it could emerge that the child was next of kin. One explained:

“*Often the child contacts me because they are struggling with something. We always talk about their home situation when they visit me. Then, it becomes clear that everything isn’t okay at home.” (Int. 6).*

The PHNs described it as important to identify children who are next of kin. They regarded themselves as dependent on others in this context and stated that they wanted to be contacted by other professionals regarding children who are next of kin: without this collaboration, they might not be able to identify the children. In the school health service, the teacher was described as one such professional, as they are able to observe changes in children and could report this to the PHN. Nevertheless, it varied from school to school or between teachers as to whether they involved the PHN in this matter. One PHN experienced a good dialog with the teachers at her school and had been contacted several times when children had parents who were ill. Others reported the opposite experience:

“*The teacher often knows that one of the parents is ill. But they seldom contact me, even if it’s in the best interest of the children.” (Int. 3).*

Consequently, in the interviews, the PHNs requested more collaboration with teachers to identify children who are next of kin.

One PHN who worked in a child health clinic described that she had identified a child as next of kin only by chance—this was in a regular meeting with the kindergarten, which had a more general focus. In this meeting, the staff from the kindergarten mentioned a child who had multiple absences because the mother had cancer. Others noted that it was a GP who had contacted them regarding further follow up for certain children. They wondered whether this lack of contact from other professionals was that those professionals believed that the PHNs already knew about the parental illness.

“*There are several parents who are ill, and nobody knows about it. Everybody thinks that others know about the ill parents. But the fact is that no one knows.” (Int. 9).*

The specialist health service also did not regularly contact the PHNs to inform them that a child was next of kin. One PHN recalled having only one such call, in her 10 years of practice:

“*I think I had one phone call from the hospital. That was [for] a recent cancer diagnosis, and they alerted me. The child wanted someone to talk to.*” *(Int. 4).*

#### Public health nurses must be observant and willing to act

All the participants in this study expressed the opinion that they should have an important role regarding children who are next of kin, although this was not always the case. Thus, they felt PHNs must be observant to cues and be willing to act, with regards to identifying a child who is next of kin.

The PHNs believed they could contribute to the children’s wellbeing simply by being an adult with whom the children could talk and from whom they could receive support. They described being able to help normalize the children’s thoughts and feelings during a difficult time. As one PHN explained:

*“Children who are next of kin have a different experience than other children. They really do. It’s important that they can get help to put it into words. It’s important to support them and try to get them to understand why they feel the way they do.” (Int. 2)*.

As parents did not always explicitly tell PHNs about their illness, the PHNs reported that they tried to be observant towards what the parents did tell them. The PHNs also described that they needed to be patient and observant in their consultations with children, to help the children open up. While it was not always evident at the outset that a child was next of kin, this information could emerge towards the end of a consultation. Often children needed time before they opened up, and the PHNs reported needing to work hard to earn the children’s trust. One PHN referred to a consultation with a 10-year-old girl:

*“I had one girl who struggled for half a year before I understood the reason. The girl had a mother with mental health problems, and she had a lot of responsibility at home. She had hardly eaten in the last half a year. She had been getting to school on her own. One day it was too much for her, so she opened up about her situation.” (Int. 2)*.

Although all the PHNs were willing to take action once they had identified a child who is next of kin, the best course of supportive action was not always clear. One PHN in a child health clinic experienced that it was difficult to support the youngest children directly. She found it easier to help the parents or professionals in the children’s network find the best ways to support the children themselves. She explained that it was more natural for young children to receive support in their normal environment, from people they know:

“*I usually support the parents and maybe the kindergarten in how they should support the child. That is a safe place for the children.” (Int. 5).*

In some cases, the PHNs found it necessary to refer a child to another professional or involve others, but they noted that it could be difficult to know when to do so. Thus, the PHNs stayed observant even after they had identified and established contact with a child who is next of kin—to be alert to changes in the child, to know when it might be necessary to involve other professionals (e.g., a teacher). One PHN recalled having involved a teacher to adapt the learning situation for a child:

*“Sometimes we have to make some changes in school. If the child is always thinking about his ill mother or father, some changes or adjustment will be good for him. At that point, it’s natural to involve the teacher and reduce the demands from school.” (Int. 10)*.

Although the PHNs in this study regarded it as part of their role to support children who are next of kin, one PHN in a child health clinic pointed to other alternatives for responsible authorities. Besides the PHN, the responsibility for children who are next of kin could also be under the remit of the children and family team, who have other and additional competences.

#### Communication is an important tool

The PHNs in this study primarily had individual consultations with children who are next of kin. They experienced that communication was the tool they most often used to support these children. However, there were no guidelines regarding the content of these communications—instead, they were based on what the PHNs believed was in line with the individual child’s needs at that time.

PHNs in the child health clinics mentioned that, until the age of five or six, the consultations were often with parents instead of the children; here, their primary role in relation to the child was to check their wellbeing in terms of healthy development.

“*One mother was depressed. So, I thought that the child might be affected. The child wasn’t able to say something so my task was to refer the mother to other professionals and to support and guide the father. I couldn’t speak directly to the child, so I just have to see if the child develops in a good way.” (Int. 7)*.

The PHNs in the school health service reported that children who are next of kin want to have consultations with the PHNs, to talk about things that were difficult to talk about when the parents were present. One PHN said:

*“I think that it’s difficult for the child who is next of kin to communicate difficult feelings and thoughts to Mom and Dad if one of them is sick. So, I’m sort of an adult person they can have this talk with.” (Int. 1)*.

PHNs used drawing as a tool to help the children communicate their feelings—this was largely used in the child health clinics and in the primary school setting. One way they used drawing was to encourage the child to colour pictures illustrating the body, identifying places where they had pain. Another way the PHNs supported children in showing their feelings was to give the feelings different colours, which helped the PHN understand the child’s feelings and initiate communication about them. Certain games were also used to get the youngest schoolchildren to talk about their feelings. One PHN referred to the ‘Hello Game’:

*“It’s about what makes you angry, and about what makes you happy. Then, we might move naturally into difficult things.” (Int. 8)*.

Sometimes it was necessary to involve other people than the parents in the consultations—for example, teachers or GPs. One PHN talked about inviting a child’s GP to a consultation so that the GP could give information to the child about the parent’s illness and prognosis. The PHN’s noted that, when they involved others, it was necessary to obtain consent from the child, but this was never a problem.

#### Follow up over time is not always provided

The PHNs emphasized the importance of ensuring follow up of children who are next of kin. Nevertheless, it varied as how this was done. Some PHNs had several consultations with a child, while others had only one. It was also mentioned that the PHNs needed to evaluate the follow up to avoid consultations without a plan, as they lacked clear guidelines and routines regarding follow up. However, when the children were visiting the PHN, a follow-up appointment was often made. The PHNs experienced that some children wanted to continue their consultations over time, while others thought it was enough to consult the PHN once and did not want any further follow up.

It also varied as to whether the PHNs followed up with just the child or with the whole family. One PHN described an instance in which she followed up with multiple members of the family, following a parent’s death:

*“So, I followed up with both the girl and her brother. I also followed up with the mother. I had a lot of follow-up meetings with them after the father died. I think it was tough but special.” (Int. 3)*.

This PHN highlighted that, for this family, it was important to follow up with the whole family, but that this was not always the case.

To provide quality follow up, PHNs underlined the importance of knowing each child’s story. They argued that the follow up must be based on each child’s needs. They also mentioned that, at times, a child would deny help. The PHN described it as difficult when being aware of a child who was struggling but did not want help. In such cases, the PHNs would try to motivate the child to come see them by describing their competences and the help they could offer, but this did not always work. One PHN described experiencing a dilemma when a father wanted his 15-year-old son to be followed up by the PHN as he was struggling after his mother’s death, but the son did not want this:

“*A 15-year-old boy who doesn’t want to talk is difficult. The father had to almost force him to come to me. We had a long, good talk and my goal was to be able to get him to come back. Unfortunately, I didn’t succeed, no matter how hard I tried.” (Int. 6)*.

It was reported as beneficial to have group consultations with children. The PHNs had positive experiences with group consultations with children of divorced parents—a clinical experience they tried to transfer to children who are next of kin. One PHN had a positive experience following up with next-of-kin siblings in a group setting. Although the PHNs called for more follow up in groups, they did note that it could be difficult to schedule the group meetings.

## Discussion

The aim of this study was to explore PHNs’ experiences working with children who are next of kin. According to the Norwegian guidelines for child health clinics and the school health service, the PHN is obliged to obtain an overview over children’s state of health [19], including children who are next of kin.

The findings indicate that the PHNs in this study largely became aware of children who are next of kin through routine consultations in the child health clinics and school health service. Sometimes it was the parents who contacted the PHN about the situation. The PHNs were concerned with how to identify children who are next of kin. They perceived their task was to have an overview of children’s state of health in the municipality. These findings are in line with a previous study [31] where it was identified that healthcare personnel generally do not identify children who are next of kin.

Children’s support needs are often neglected by parents and healthcare personnel [22]. Despite this risk, the PHNs in this study described a lack of guidelines and routines for identifying these children and their needs. This is problematic as the PHNs are to monitor, identify, guide, counsel and refer children to other professionals when needed, and to do that they need to know how to proceed. It has been reported that nurses have concerns about caring for children who are next of kin, due to feelings of uncertainty and not daring to face these children. These concerns increase when the nurses feel they lack knowledge and experience [24, 31], which support the notion that guidelines and routines are needed to be in place for the PHNs to better care for children who are next of kin and counteract the risk that the children’s needs are neglected.

The Norwegian legislation intended that designated practitioners in hospitals and specialist health services should initiate and establish collaboration with professionals within the municipalities [1]. The Norwegian guidelines for PHNs emphasize collaboration with other professionals to identify and follow up children who are next of kin [19], something also mentioned in the current study. However, the PHNs in the study did not reflect on whose responsibility it was to initiate collaboration. Collaboration is described as one of the cornerstones of the PHN profession [32]. Nevertheless, the PHNs in this study pointed to a lack of routines and guidelines related to collaboration with other professionals, and they did not describe having any own rule of the thumb when to initiate contact, nor with whom. Instead, they described the need to be observant and use their competence to decide from case to case when it was necessary to involve other professionals. In some cases, they did collaborate with other professionals, but this collaboration was largely coincidental. A previous study has highlighted the importance of interprofessional collaboration around children who are next of kin; the authors found that established structures for collaboration were needed for optimal care for both children and parents [13], which seems to be the case also in the current settings. The PHNs in the present study requested more collaboration, especially with teachers but also other professionals, which is an important issue for stakeholders to take into consideration when organizing sustainable support for children who are next of kin. In addition, the study findings suggest that there is often a lack of interaction between the specialist and municipal health services around the follow up of children who are next of kin, which could be another area for improvement to ensure that children who are next of kin receive the necessary support [13].

As mentioned earlier, in Norway, legislation stipulates that the specialist health service designates one practitioner in each department to ensure follow up of children who are next of kin [17, 18], but this is not required by law in municipal care. Doing so, however, might increase the likelihood that children who are next of kin will be identified and followed up systematically. It might also enhance interprofessional collaboration across healthcare levels by clarifying the point of contact. As the PHN has a statutory responsibility for children in municipalities, they might in the future be well positioned to fill such a role. However, the findings indicate that before such an implementation more regulations, guidelines and routines need to be in place.

Findings indicate that, as PHNs do not always know about each child’s family situation, they must be observant to cues during their conversations with both children and parents. This is supported by research showing that it is important that healthcare personnel be aware when parents are ill, as otherwise important information about the child can be missed [25]. However, when the PHNs had consultations with children, it was not always clear when they were next of kin. There could be various explanations for this. A child might not explicitly reveal that they are next of kin in cases of parental mental illness—the child may feel shame when their parent behaves differently, preferring to conceal their family life to avoid stigmatisation [12]. A child might also not initially disclose that they are next of kin because they are testing whether they can trust the PHN. This has been reported in a previous study, which found that children sometimes visit PHNs for physical injuries to check whether they can trust them [33].

The PHNs in this study pointed to communication as the most commonly used tool when supporting school-aged children who are next of kin. The PHNs typically had individual consultations with the children and, while they felt that these conversations were in line with each child’s needs, they pointed to a lack of guidelines regarding the content of such visits. However, previous research does suggest that communicating with children about their parent’s illness is important [24]. Studies indicate that children who are next of kin need someone to talk to about their feelings, problems and parent’s diagnosis; moreover, this person needs to be able to listen and understand, and to be encouraging and reassuring [34]. It has been found that children view the communication with the school nurse as an opportunity to discuss their own health and situation [35], which provides further evidence in support of the PHNs’ use of communication as a tool.

Communicating with children about their parent’s illness may reduce anxiety about what is happening in the family [14]. Furthermore, school-aged children are likely used to talking to the school nurse, as they regularly take part in health dialogs, where they are given the opportunity to discuss their own health and situation [35]. However, a Norwegian study found that adolescents with mental health problems only use the school health service to a limited extent, and boys even less so than girls [36]. To effectively reach children, PHNs must be readily available [33, 37], be able to collaborate around children with problems [33] and use competences and skills to identify those problems [38].

The PHNs in our study reported having positive experiences with following up children who are next of kin (including siblings) in groups, although scheduling group sessions could be difficult. According to the national guidelines, follow-up groups are recommended as a tool that can help identify and categorize children’s needs for follow up [19]. Groups may be a way for children who are next of kin to meet others who are in a similar situation. Evidence supporting this approach is found in previous research: for example, studies showing that peers who have similar experiences (e.g. having parents with a similar illness) are better able to understand what each other are going through and can offer support in a number of ways [39, 40]. Moreover, during group sessions, children can learn how to better manage relationships that are important to them and can benefit from learning how to talk about their experiences with those whom they value [41]. One study identified that, by participating in groups and sharing their experiences, children feel a genuine sense of connection; they also gain information from their peers about how others have tackled the situation [39]. Support groups does not necessarily need to be run by nurses, rather, according to a recent scoping review such groups in the municipality setting was seldom run by nurses [34]. Nevertheless, depending on how the health care is organized in different settings, it is reasonable that PHN could run support groups, perhaps in collaboration with other professionals or with representatives from nongovernmental organizations.

### Methodological discussion

The trustworthiness of this study was guided by the concepts defined by Lincoln and Guba [42] and also the descriptions of Granheim and Lundman [27] related to credibility, transferability, dependability and confirmability. Credibility was ensured by including participants representing a variation in age, educational level, length of work experience and number of children who are next of kin with whom they followed up. In the analysis, credibility was ensured via continuous dialogue between the researchers to reach consensus regarding the content and labelling of the categories, to ensure that these were in accordance with the content of the interviews. Dependability was ensured through consistency in the conducting of the interviews. One of the researchers conducted the interviews, using a semi-structured interview guide. Confirmability was ensured in several ways: The first author read and reread the transcripts several times, and the other authors read and reread parts of the transcribed materials to ensure that the categories were in line with the transcripts and to avoid having author preconceptions colouring the interpretation. Several quotations from the interviews are presented to illustrate the content of the categories, and to enable the reader to judge the confirmability of the findings [27].

One limitation might be the relatively small number of PHNs in the study sample due to the PHNs’ extended workload related to the COVID-19 pandemic. Nevertheless, the interviews represent PHN from both child health clinics and school health service and contributed to a rich amount of data. Another limitation is that the PHNs were all women—though this is representative, as there are few male PHNs in Norway. Moreover, all of the participants had been working for at least four years, representing an additional limitation: Including younger and more recently educated PHNs might have contributed to further variation in the findings, as there is growing awareness in government documents and nursing/PHN education that PHNs should be observant regarding children who are next of kin.

## Conclusion

Irrespectively of working in a child health clinic or in school health service, the PHNs experienced uncertainty concerning how to identify and follow up with children who are next of kin but were observant and willing to act in the best interest of these children. They drew on their skills and competences when improvising support, with the child’s best interest as their main focus. When striving for the best interest of the child, the PHNs were at times dependent on collaboration with other professionals, as well as the children’s parents, to achieve their objectives. Although they were acting on their own initiative, they emphasized the need for guidelines around this particular responsibility and role.

### Relevance to clinical practice

This study adds to the international knowledge about support to children who are next of kin. It offers insights about how PHN in the municipality experience their work with this group. The knowledge provided by the current study offers valuable insight into strengths and limitations in the support for children who are next of kin and could inform stakeholders in organizing sustainable support for this group. Findings demonstrate that, despite the lack of regulations regarding those responsible for children who are next of kin in municipal care, the PHNs do provide care for them—though their identification as next of kin and their follow up is largely coincidental. Thus, the authors argue that there is a need for a more systematic follow up for children who are next of kin in the municipalities, safeguarded by legislation, guidelines and routines. Study findings offer support for the notion that the PHN could take on a designated role in municipal care to ensure follow up of children who are next of kin, similar to the mandate in the Norwegian specialist health service.

## Data Availability

The datasets generated and/or analysed during the current study are not publicly available due to the protection of the anonymity of the participants but are available from the corresponding author on reasonable request.

## Abbreviations

GP: General practitioner
PHN: Public health nurse

## References

[1] Lauritzen C, Reedtz C (2016). Child responsible personnel in adult mental health services. Int J Ment Health Syst.

[2] Kavanaugh MS, Kavanaugh MS, Stamatopoulos V, Stamatopoulos V, Cohen D, Cohen D, Zhang L, Zhang L (2016). Unacknowledged caregivers: a scoping review of Research on Caregiving Youth in the United States. Adolesc Res Rev.

[3] Leu A, Becker S (2017). A cross-national and comparative classification of in-country awareness and policy responses to ‘young carers’. J Youth Stud.

[4] Ruud T, Birkeland B, Faugli A, Hagen KA, Hellman A, Kallander EK, Kufås E, Løvås M, Peck GC, Skogerbø, Å, Skogøy BE, Stavnes K, Thorsen E, Weimand BM: Barn som pårørende - Resultat fra en multisenterstudie [Children as next of kin - Result from a multicenterstudy IS-0522. In.: Akershus Universitetssykehus; 2015.

[5] Appel CW, Frederiksen K, Hjalgrim H, Dyregrov A, Dalton SO, Dencker A, Høybye MT, Dige J, Bøge P, Mikkelsen OA (2019). Depressive symptoms and mental health-related quality of life in adolescence and young adulthood after early parental death. Scand J Public Health.

[6] Drost LM, Krieke L, Sytema S, Schippers GM (2016). Self-expressed strengths and resources of children of parents with a mental illness: a systematic review. Int J Ment Health Nurs.

[7] Huang X, O’Connor M, Lee S (2014). School-aged and adolescent children’s experience when a parent has non-terminal cancer: a systematic review and meta-synthesis of qualitative studies. Psychooncology.

[8] Torvik FA, Rognmo K (2011). Barn av foreldre med psykiske lidelser eller alkoholmisbruk. Omfang og konsekvenser. Rapport Nasjonalt folkehelseinstitutt.

[9] Gullbra F, Smith-Sivertsen T, Graungaard AH, Rortveit G, Hafting M (2016). How can the general practitioner support adolescent children of ill or substance-abusing parents? A qualitative study among adolescents. Scand J Prim Health Care.

[10] Dam K, Joensen DG, Hall EOC (2018). Experiences of adults who as children lived with a parent experiencing mental illness in a small-scale society: a qualitative study. J Psychiatr Ment Health Nurs.

[11] Ireland MJ, Pakenham KI (2010). Youth adjustment to parental illness or disability: the role of illness characteristics, caregiving, and attachment. Psychol Health Med.

[12] Dam K, Hall EO (2016). Navigating in an unpredictable daily life: a metasynthesis on children’s experiences living with a parent with severe mental illness. Scand J Caring Sci.

[13] Golsäter M, Knutsson S, Enskär K. Children’s experiences of information, advice and support from healthcare professionals when their parent has a cancer disease-experiences from an oncological outpatient department. Eur J Oncol Nurs. 2021;50:101893. 10.1016/j.ejon.2020.101893 33465701

[14] Fearnley R, Boland JW (2017). Communication and support from health-care professionals to families, with dependent children, following the diagnosis of parental life-limiting illness: a systematic review. Palliat Med.

[15] Thomas N, Stainton T, Jackson S, Cheung WY, Doubtfire S, Webb A (2003). Your friends don’t understand’: invisibility and unmet need in the lives of ‘young carers. Child Fam Soc Work.

[16] Joseph S, Sempik J, Leu A, Becker S (2020). Young carers research, practice and policy: an overview and critical perspective on possible future directions. Adolesc Res Rev..

[17] Spesialisthelsetjenesteloven [Specialist Health Service. Act]. LOV-1999-07-02-61 https://lovdata.no/dokument/NL/lov/1999-07-02-61.

[18] Helsepersonelloven [HealthP, Act]. LOV-1999-07-02-64 https://lovdata.no/dokument/NL/lov/1999-07-02-64.

[19] Norwegian Directorate of Health. Nasjonal faglig retningslinje i det helsefremmende og forebyggende arbeidet i helsestasjon, skolehelsetjenesten og helsestasjon for ungdom [National guidelines for health promoting work in health care sevice, school health service and youth health services]. 2017.

[20] Kallander EK, Weimand B, Ruud T, Becker S, Van Roy B, Hanssen-Bauer K (2018). Outcomes for children who care for a parent with a severe illness or substance abuse. Child Youth Serv.

[21] Kallander EK, Weimand BM, Becker S, Van Roy B, Hanssen-Bauer K, Stavnes K, Faugli A, Kufas E, Ruud T (2018). Children with ill parents: extent and nature of caring activities. Scand J Caring Sc.

[22] Martinsen EH, Weimand BM, Pedersen R, Norvoll R (2019). The silent world of young next of kin in mental healthcare. Nurs Ethics.

[23] Mahoney L (2010). Children living with a mentally ill parent: the role of public health nurses. Nurs Prax N Z.

[24] Golsäter M, Henricson M, Enskär K, Knutsson S (2016). Are children as relatives our responsibility? – how nurses perceive their role in caring for children as relatives of seriously ill patients. Eur J Oncol Nurs.

[25] Gullbrå F, Smith-Sivertsen T, Rortveit G, Anderssen N, Hafting M (2014). To give the invisible child priority: children as next of kin in general practice a qualitative study among general practitioners. Scand J Prim Health Care.

[26] Beatriz MB, Iris Del Mar LG (2017). VIVA caregivers: proposal of a methodology for social inclusion of female caregivers. Eur J Physiother.

[27] Graneheim UH, Lundman B (2004). Qualitative content analysis in nursing research: concepts, procedures and measures to achieve trustworthiness. Nurse Educ Today.

[28] Tong A, Sainsbury P, Craig J (2007). Consolidated criteria for reporting qualitative research (COREQ): a 32-item checklist for interviews and focus groups. Int J Qual Health Care.

[29] FOR-2018-10-19-1584 (2018). Forskrift om kommunens helsefremmende og forebyggende arbeid i helsestasjons- og skolehelsetjenesten.

[30] Granrud MD, Anderzèn-Carlsson A, Bisholt B, Steffenak AKM (2019). Public Health Nurses’ perceptions of interprofessional collaboration related to adolescents’ mental health problems in secondary schools: a phenomenographic study. J Clinl Nurs.

[31] Knutsson S, Enskär K, Golsäter M (2017). Nurses’ experiences of what constitutes the encounter with children visiting a sick parent at an adult ICU. Intensive Crit Care Nurs..

[32] Glavin K, Schaffer MA, Halvorsrud L, Kvarme LG (2014). A comparison of the Cornerstones of Public Health nursing in Norway and in the United States. Public Health Nursing.

[33] Granrud MD, Bisholt B, Anderzèn-Carlsson A, Steffenak AKM (2020). Overcoming barriers to reach for a helping hand: adolescent boys’ experience of visiting the public health nurse for mental health problems. Int J Adolesc Youth.

[34] Steffenak AKM, Anderzén-Carlsson A, Opheim E, Sandsdalen T (2021). Community-based support for children who are next-of-kin for a parent experiencing illness or disability–a scoping review. BMC Health Serv Res.

[35] Rising Holmström M, Boström L (2021). Student perspectives on health dialogues: how do they benefit?. Int J Qualitative Stud Health Well-being.

[36] Granrud MD, Steffenak AKM, Theander K (2017). Gender differences in symptoms of depression among adolescents in Eastern Norway: results from a cross-sectional study. Scand J Public Health..

[37] Bains RM, Diallo AF (2016). Mental Health Services in School-Based Health Centers. J School Nurs.

[38] Bohnenkamp JH, Stephan SH, Bobo N (2015). Supporting student mental health: the role of the school nurse in coordinated school mental health care. Psychol Sch.

[39] Maynard A, Patterson P, McDonald FE, Stevens G (2013). What is helpful to adolescents who have a parent diagnosed with cancer?. J Psychosoc Oncol.

[40] Templeton L, Novak C, Wall S (2011). Young people’s views on services to help them deal with parental substance misuse. Drugs: Educ Prev policy.

[41] Gladstone BM, McKeever P, Seeman M, Boydell KM (2014). Analysis of a support group for children of parents with mental illnesses: managing stressful situations. Qual Health Res.

[42] Lincoln YS, Guba EG (1986). But is it rigorous? Trustworthiness and authenticity in naturalistic evaluation. New Directions for Evaluation..

[43] Northern Nurses Federation (2003). Etiska riktlinjer för omvårdnadsforskning i Norden [Ethical guidelines for nursing research in the nordic countries]. Vard Nord Utveckl Forsk.

